# Enzymatic Properties of *Populus* α- and β-NAD-ME Recombinant Proteins

**DOI:** 10.3390/ijms140712994

**Published:** 2013-06-24

**Authors:** Jinwen Liu, Qiguo Yu, Nabil I. Elsheery, Yuxiang Cheng

**Affiliations:** 1 State Key Laboratory of Tree Genetics and Breeding, Northeast Forestry University, 26 Hexing Road, Harbin 150040, China; E-Mails: jin-wenliu@163.com (J.W.); yuqiguo1989@hotmail.com (Q.Y.); 2 College of Life Science and Technology, Heilongjiang Bayi Agricultural University, Daqing 163319, China; 3 Agricultural Botany Department, Faculty of Agriculture, Tanta University, Tanta 31527, Egypt; E-Mail: nshery@yahoo.com

**Keywords:** NAD-malic enzyme, *Populus trichocarpa*, glutathione S-transferase fusion protein, enzymatic property

## Abstract

Plant mitochondrial NAD-malic enzyme (NAD-ME), which is composed of α- and β-subunits in many species, participates in many plant biosynthetic pathways and in plant respiratory metabolism. However, little is known about the properties of woody plant NAD-MEs. In this study, we analyzed four *NAD-ME* genes (*PtNAD-ME1* through *PtNAD-ME4*) in the genome of *Populus trichocarpa*. PtNAD-ME1 and -2 encode putative α-subunits, while PtNAD-ME3 and -4 encode putative β-subunits. The *Populus* NAD-MEs were expressed in *Escherichia coli* cells as GST-tagged fusion proteins. Each recombinant GST-PtNAD-ME protein was purified to near homogeneity by glutathione-Sepharose 4B affinity chromatography. Milligram quantities of each native protein were obtained from 1 L bacterial cultures after cleavage of the GST tag. Analysis of the enzymatic properties of these proteins *in vitro* indicated that α-NAD-MEs are more active than β-NAD-MEs and that α- and β-NAD-MEs presented different kinetic properties (*V*_max_, *k*_cat_ and *k*_cat_/*K*_m_). The effect of different amounts of metabolites on the activities of *Populus* α- and β-NAD-MEs was assessed *in vitro*. While none of the metabolites evaluated in our assays activated *Populus* NAD-ME, oxalacetate and citrate inhibited all α- and β-NAD-MEs and glucose-6-P and fructose inhibited only the α-NAD-MEs.

## 1. Introduction

Malic enzyme (ME) catalyzes the oxidative decarboxylation of malate to pyruvate and CO_2_ in the presence of a divalent metal ion using NAD or NADP as a cofactor. The products of the reaction participate in many biosynthetic pathways and in respiratory metabolism [1]. MEs are classified into three different groups. The first includes the NAD-malic enzymes (NAD-MEs, EC 1.1.1.38) found in *Ascaris suum* and bacteria, which use NAD and can decarboxylate oxaloacetate (OAA) in addition to malate. The second group consists of the plant NAD-MEs (EC 1.1.1.39), which are not able to decarboxylate OAA. A third group includes the NADP-malic enzymes (NADP-MEs, EC 1.1.1.40). Plant NAD-ME isoforms are found in the mitochondria [2]. NAD-MEs in some C4 and CAM plants provide CO_2_ for the Calvin cycle during photosynthetic metabolism [3–5]. Furthermore, malate is decarboxylated in the mitochondria through the action of NAD-MEs to produce pyruvate, which is oxidized in the tricarboxylic acid (TCA) cycle [1,5]. Recent metabolic profiling of Arabidopsis plants completely lacking NAD-ME activity revealed that NAD-MEs have a major influence over nocturnal metabolism [6].

Plant NAD-MEs have been found to be composed of two dissimilar subunits (α- and β-) in some species such as potato (*Solanum tuberosum*; C_3_ plant) [7], *Urochloa panicoides* (C4-PEPCK plant) [8], *Crassula argentea* (CAM plant) [9], and *Amaranthus hypochondriacus* (C4-NAD-ME plant) [10]. The α- and β-subunits have similar primary amino acid sequences [2] and *in vitro* studies have provided evidence that both α- and β-subunits are required for NAD-ME activity [9,11]. Another study suggested that the β-subunit plays a regulatory role in NAD-ME activity [10]. However, recent studies showed that the separated recombinant proteins of *Arabidopsis thaliana* (AtNAD-ME1 and -2) showed NAD-ME activity and display distinct kinetic mechanisms [6,12].

The genes encoding the α- and β-subunits of plant NAD-MEs were identified from various plants, including potato, amaranth and *Arabidopsis* [6,10,13]. Plant α- and β-NAD-ME are encoded by single genes in the species analyzed so far. NAD-MEs assemble as heterodimers, heterotetramers or heterooctamers in some species, while it forms a homomer in others [6,12,14]. Some plant NAD-MEs are activated by fumarate and coenzyme A (CoA) [15,16] and are also potentially regulated via changes in aggregation state [7,11]. Little is known about the properties of NAD-MEs in woody plants. In this study, we characterized two putative α-NAD-MEs (PtNAD-ME1 and -2) and two putative β-NAD-MEs (PtNAD-ME3 and -4) of the woody model plant. The proteins were successfully expressed in *E. coli* cells as fusion proteins. The purified recombinant proteins were used to investigate their enzymatic properties.

## 2. Results and Discussion

### 2.1. Characterization and Expression Pattern of the PtNAD-ME Gene Family from *Populus*

**Figure 1:**
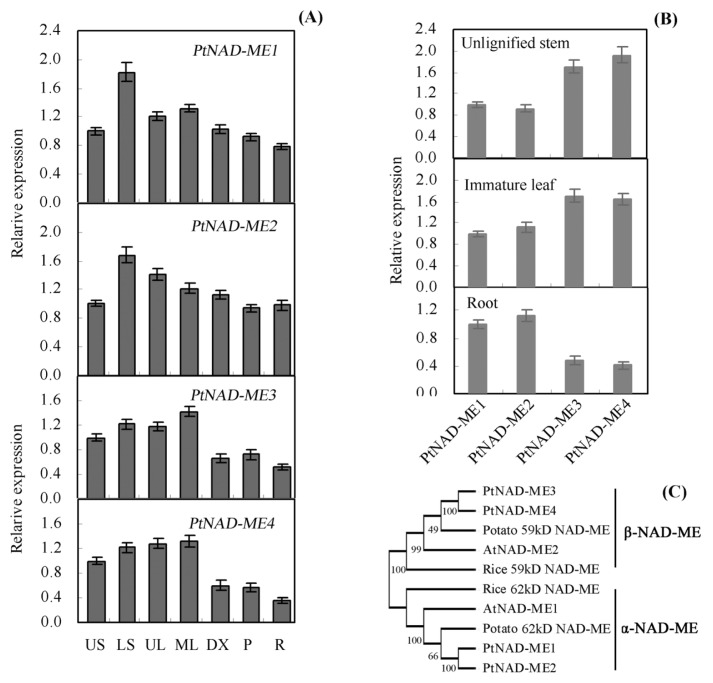
Characterization of the *PtNAD-ME* gene family in *Populus*. (**A**) Analysis of *PtNAD-ME* gene expression in *Populus* by real-time PCR. US, unlignified stem (internodes 1–3 from top); LS, lignified stem (from internodes 8–10); UL, immature leaf (from internodes 2–3); ML, mature leaf (from internodes 8–10); DX, developing xylem (from the basal internodes); Phloem (P, from the basal internodes); R, Root; (**B**) Comparison of four *PtNAD-ME* gene expression levels in unlignified stem, immature leaf, and root tissues of *Populus*, respectively; (**C**) Phylogenetic analysis of the *Populus* NAD-MEs with other NAD-MEs by neighbor joining method using MEGA 4.0 software. Accession numbers and sources of the NAD-MEs are as follows: AtNAD-ME1 (At2g13560) and AtNAD-ME2 (At4g00570) from Arabidopsis, rice 59 and 62 kDa NAD-MEs (LOC_Os07g31380 and LOC_Os10g35960 ) and potato 59 and 62 kDa NAD-MEs.

Using the sequences of *Arabidopsis* NAD-MEs (At2g13560 and At4g00570) [6], four *NAD-ME* genes were found in the genome of *Populus* (http://genome.jgi-psf.org/Poptr1_1/Poptr1_1.home.html). These genes were named *PtNAD-ME1*, *PtNAD-ME2*, *PtNAD-ME3*, and *PtNAD-ME4* (Table S1). RT-PCR and DNA sequencing analysis showed that these four *PtNAD-MEs* are all expressed genes in *Populus*. We further validated their full-length CDS in the *Populus* genome database (data not shown). We examined transcript levels in the different *Populus* tissues by quantitative real-time PCR. The genes *PtNAD-ME1*, *PtNAD-ME2*, *PtNAD-ME3* and *PtNAD-ME4* were expressed in various tissues (Figure 1A). Compared with their relative transcript levels, *PtNAD-ME1* and *PtNAD-ME2* have transcript levels similar to one another in unlignified stem, immature leaf, and root tissues. The corresponding transcript levels of *PtNAD-ME3* and *PtNAD-ME4* are also similar (Figure 1B). In root tissue, the transcript levels of *PtNAD-ME1* and *PtNAD-ME2* were higher than those of *PtNAD-ME3* and *PtNAD-ME4* (Figure 1B). To gain further insight into the structural diversity of *PtNAD-ME* genes, we compared the exon/intron organization and showed that PtNAD-ME1 and -2 and PtNAD-ME3 and -4 share similar exon/intron structure, respectively (Figure S1). The amino acid sequence analysis revealed high identity among the PtNAD-ME families: 96% identity for PtNAD-ME1 *versus* PtNAD-ME2 and 92% identity for PtNAD-ME3 *versus* PtNAD-ME4 (Table S2 and Figure S2). The phylogenetic tree, constructed with the neighbor joining (NJ) method using the program MEGA 4.0 [17], indicated that PtNAD-MEs are divided into two clades (Figure 1C). Some plant NAD-MEs are composed of α- and β-subunits, with molecular masses of approximately 62 and 58 kDa, respectively [9]. PtNAD-ME1 and PtNAD-ME2 belong to the α-NAD-MEs, and PtNAD-ME3 and PtNAD-ME4 clustered with β-NAD-MEs. All *Populus* NAD-MEs are predicted to contain a mitochondrial targeting peptide using TargetP (http://www.cbs.dtu.dk/services/TargetP) and SignalP (http://www.cbs.dtu.dk/services/SignalP). To the best of our knowledge, only one gene encoding α-NAD-ME or β-NAD-ME exists in *Arabidopsis*, potato, *Urochloa panicoides*, and *Crassula argentea*, while the enzymes isolated from *Eleusine coracana*, *Panicum dichotomiflorum*, or *Amaranthus tricolor* were postulated to be composed of identical subunits [6–8,10,18,19].

### 2.2. Expression and Purification of PtNAD-ME Proteins in *E. coli*

**Figure 2:**
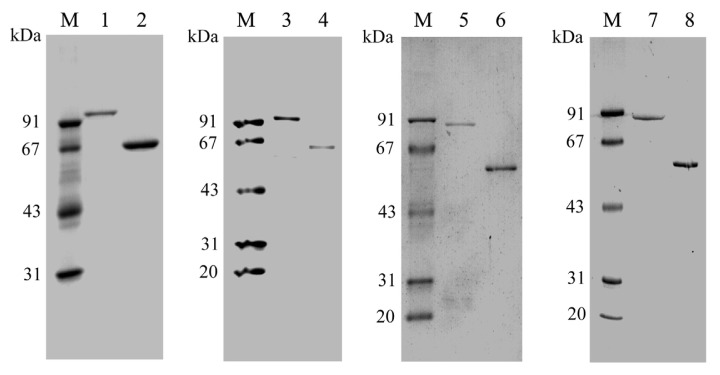
SDS-PAGE analysis of the recombinant *Populus* NAD-MEs purified from *E. coli* BL21 cells. M, marker; 1–2, GST-PtNAD-ME1 (1 μg) and PtNAD-ME1 (5 μg); 3–4, GST-PtNAD-ME2 (2 μg) and PtNAD-ME2 (1 μg); 5–6, GST-PtNAD-ME3 (1 μg) and PtNAD-ME3 (2 μg); 7–8, GST-PtNAD-ME4 (1 μg) and PtNAD-ME4 (2 μg).

To confirm the proposed function of the putative *PtNAD-ME* genes, the coding regions of each mature PtNAD-ME were amplified by PCR without signal peptide sequences, cloned in pGEX-6p-3 vector and heterologously expressed in *E. coli* BL21 cells under the control of the T7 promoter. After 2 h induction with 1 mM IPTG, the presence of each PtNAD-ME fusion protein with the GST tag increased significantly, as determined by SDS-PAGE (Figure S3). The molecular masses of GST-PtNAD-ME1 and GST-PtNAD-ME2 fusion proteins with the 26 kDa GST tag were approximately 93 and 92 kDa, respectively, and approximately 87 kDa for GST-PtNAD-ME3 and GST-PtNAD-ME4 (Figure 2).

Under optimal growth conditions, the cells were collected from 1 L bacterial cultures and lysed with lysozyme. The recombinant active GST-tagged PtNAD-ME proteins (GST-PtNAD-ME1 to GST-PtNAD-ME4) were purified in a single step to near homogeneity by glutathione-Sepharose 4B affinity chromatography (Figure 2). After cleavage of the GST tags using PreScission protease, the molecular masses of native proteins of PtNAD-ME1 to PtNAD-ME4 were approximately 67, 66, 61, and 61 kDa on SDS-PAGE gels, respectively (Figure 2). The results of PtNAD-ME purification are summarized in Table 1.

The purified recombinant PtNAD-ME1 and -2 (α-NAD-MEs) have high specific activities of 34.0 U/mg and 40.5 U/mg, respectively. However, the activities of PtNAD-ME3 and -4 (β-NAD-MEs) are very low (Table 1). Previous reports showed that in some plant species both α- and β-subunits are required for NAD-ME activity [9–11]. Taken together, our data suggest that *Populus* NAD-ME might be composed of dissimilar subunits where the α-subunit would contribute to catalysis and the β-subunit may have a regulatory role. However, Arabidopsis α- and β-subunit proteins displayed high NAD-ME activity and distinct kinetic mechanisms [6,12].

### 2.3. Optimum pH of Recombinant PtNAD-ME Proteins

**Figure 3:**
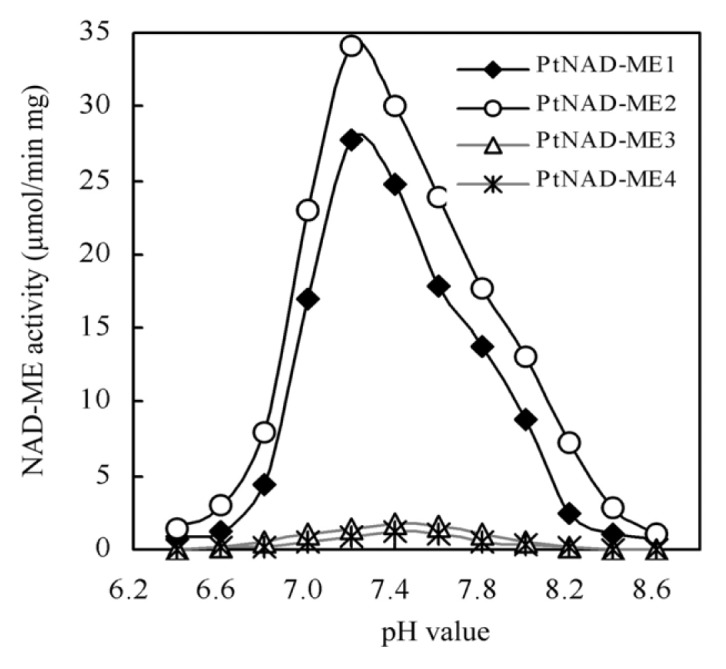
Effect of pH on the activity four purified PtNAD-ME recombinant proteins. The activity of NAD-ME was determined from measurements of the oxidative decarboxylation of malate as described by Tronconi *et al.* [6]. Reactions were started by the addition of purified PtNAD-ME recombinant proteins. Values indicate the means of three independent measurements.

The effect of pH on the activity of each recombinant PtNAD-ME protein was investigated. Specific activity was determined from measurements of the oxidative decarboxylation of malate at different pH values (6.2–8.6). When the pH values ranged between 7.0 and 7.8, each recombinant PtNAD-ME enzyme retained more than 60% activity. The optimum pH values of the *Populus* α-and β-NAD-MEs were 7.2 and 7.4, respectively (Figure 3). For comparison, the optimal pH values for purified NAD-MEs from other plants are reported to be 7.2 in the CAM plant *Mesembryanthemum crystallinum* [4], 6.4 to 6.6 in the C_3_ plant *Arabidopsis* [6] and 7.4 in the C_4_ plant *Panicum miliaceum* [20]. The optimal pH values for *Populus* NAD-MEs are similar to those of *Panicum miliaceum* and *Mesembryanthemum crystallinum*.

### 2.4. Kinetic Parameters for Recombinant PtNAD-ME Proteins

**Figure 4:**
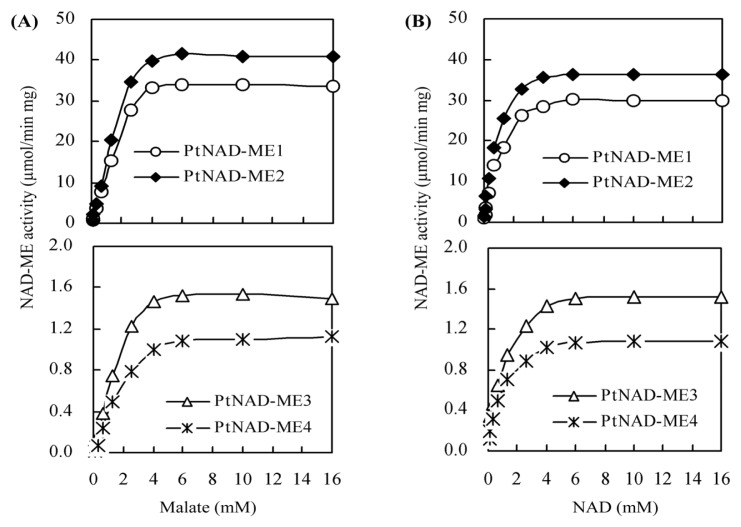
Activity curves of recombinant PtNAD-ME. Assays were performed at the optimum pH of each PtNAD-ME. The results are presented as the mean of triplicate determinations.

Kinetic reaction of the recombinant *Populus* α- and β-NAD-MEs were determined by varying the concentrations of one substrate while keeping the concentrations of other substrates and the cofactor at saturation levels. When malate was the limiting substrate (0.008–16 mM), the activities of α-NAD-MEs (PtNAD-ME1 and -2) and β-NAD-MEs (PtNAD-ME3 and -4) were shown in Figure 4A. The result showed that high concentrations of malate (from 5 to 16 mM) inhibited neither α- nor β-NAD-MEs. Similarly, the activities of *Arabidopsis* recombinant NAD-ME1 and NAD-ME2 (α- and β-NAD-MEs) are unaffected at a saturating concentration of malate [12,14]. When NAD was the limiting substrate (0.008–16 mM) and the saturated concentration of malate was 4 mM, the activities of *Populus* α- and β-NAD-MEs were shown in Figure 4B.

Kinetic parameters were determined for the recombinant *Populus* NAD-MEs (Table 2). The kinetic properties of both α-NAD-MEs were comparable. In addition, the kinetic properties of both β-NAD-MEs were similar. The α- and β-NAD-MEs showed different kinetic properties. The velocities of the reactions *(V*_max_ and *k*_cat_) catalyzed by the α-NAD-MEs were approximately 20 times higher than those catalyzed by the β-NAD-MEs. In addition, the *K*_m_ values for malate and NAD were in the same order for all PtNAD-MEs. The efficiencies of the reactions catalyzed by the α-NAD-MEs were thus much higher than those of the β-NAD-MEs (Table 2).

### 2.5. Effect of Catabolites on the Activities of Recombinant PtNAD-MEs

**Figure 5:**
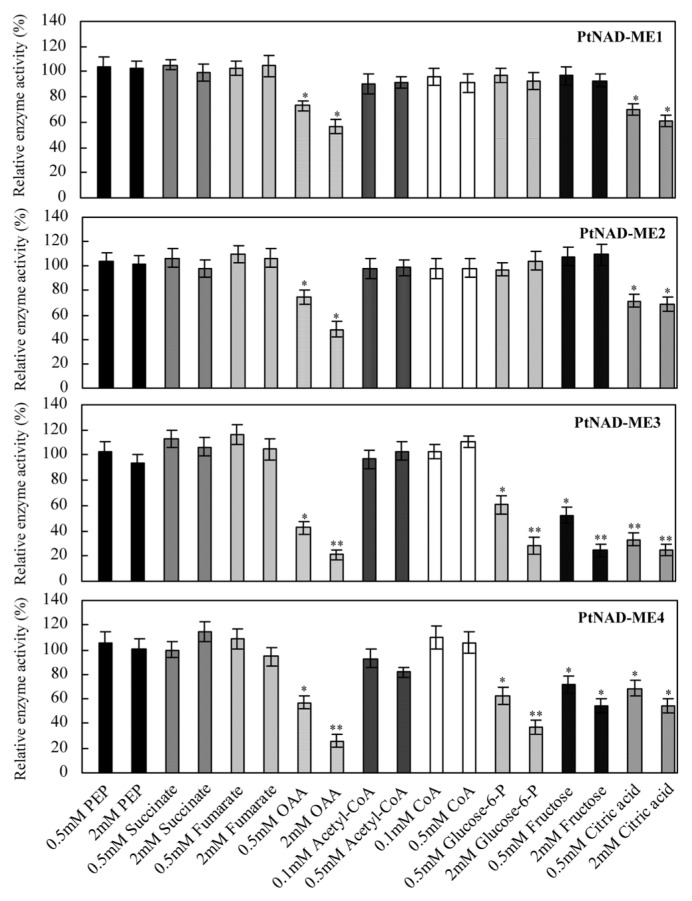
Effect of different metabolites on the activity of each purified PtNAD-ME recombinant proteins. Activities were measured at the optimum pH of each PtNAD-ME in the absence or presence of each effector. The results represent the percent of activity in the presence of each effector relative to the activity measured in the absence of the metabolites (100%, control). Values are presented as the mean of triplicate determinations. * and ** differ significantly from the control with *p* < 0.05 and *p* < 0.01, respectively, by Student’s *t-*test.

The activities of recombinant *Populus* α- and β-NAD-ME proteins in the malate decarboxylation reaction were evaluated in the presence of different metabolites from mitochondrial respiration, the tricarboxylic acid cycle and glycolytic metabolism (Figure 5). Among the metabolites assayed, OAA and citrate inhibited the activities of all á- and â-NAD-MEs. In addition, glucose-6-P and fructose also inhibited the â-NAD-MEs. The plant mitochondrial NAD-ME enzyme has a key role in mitochondrial carbon metabolism, providing a means whereby organic acids can be partitioned between replenishment of mitochondrial pools and complete oxidation [13]. None of the metabolites evaluated in this assay activated *Populus* NAD-MEs. However, *Arabidopsis* NAD-ME1 was strongly activated by OAA and fumarate while PEP, FBP, acetyl-CoA and CoA enhanced the activity of NAD-ME2 [14].

## 3. Experimental Section

### 3.1. Plant Growth

*Populus trichocarpa* was grown in a greenhouse under long day conditions (16 h light/8 h dark) at temperatures of 25–28 °C. Unlignified stem (internodes 1–3 from top), lignified stem (from internodes 8–10), immature leaf (from internodes 2–3), mature leaf (from internodes 8–10), terminal bud, developing xylem (from the basal internodes), phloem (from the basal internodes) and root tissues were separately harvested from one-year-old trees. All samples were immediately frozen in liquid nitrogen and stored at −80 °C before the isolation of total RNA.

### 3.2. Isolation of PtNAD-ME cDNAs and Real-Time PCR Analysis

Total RNA was isolated from 0.2 g of different tissues of *Populus* using the pBIOZOL plant total RNA Extraction Reagent according to the manufacturer’s instructions (BioFlux, Hangzhou, China). The quantity and purity of isolated total RNA were determined spectrophotometrically, and the integrity of the RNA was verified by 1.5% denaturing formaldehyde agarose gel electrophoresis. Total cDNAs were synthesized with RNA LA PCR^™^ Kit (AMV) Ver.1.1 (Takara Company, Dalian, China). Full-length sequences of *Populus NAD-ME* genes were obtained by RT-PCR (the primers used are described in Table S3) using total cDNAs as templates, subcloned into the entry vector pENTR/D-TOPO (Invitrogen, Carlsbad, NM, USA) and completely sequenced. Real-time PCR analysis was performed to determine the expression levels of the four *PtNAD-ME* genes in different *Populus* tissues. The pairs of primers used are described in Table S4.

### 3.3. Construction of Expression Plasmids

The coding regions of each mature PtNAD-ME (without signal peptide sequences) were amplified using the primers shown in Table S5. The PCR products were ligated into the vector pGEX-6p-3 after it had been treated with the appropriate restriction enzymes. The resulting plasmids were confirmed by DNA sequencing and were named pGEX-6p-3-PtNAD-MEs. *E. coli* BL21 cells with individual pGEX-6p-3-PtNAD-ME plasmids were used to express the recombinant PtNAD-ME proteins fused with GST tags.

### 3.4. Expression and Purification of *Populus* PtNAD-ME Proteins in *E. coli*

BL21 cells were cultured overnight in LB medium containing 100 μg/mL ampicillin at 37 °C, diluted 1:100 with fresh pre-warmed LB medium containing ampicillin and incubated at 28 °C with shaking at 150 rpm. When OD_600_ had reached a value of 1.0, expression of GST-PtNAD-ME was induced by addition of IPTG to a final concentration of 0.1 mM, and the cells were incubated for an additional 6 h. The cells were pelleted by centrifugation at 6000× *g* for 5 min. Cell pellets were resuspended in a pre-cooled lysis buffer (150 mM NaC1, 50 mM Tris-HCl, pH 8.0, 1 mM EDTA, 1 mM DTT, and 1 mM PMSF), and lysozyme was added to the suspension to a final concentration of 1 mg/mL. The suspension was incubated on ice for 1 h and centrifuged at 40,000× *g* for 30 min. The supernatant, containing the fusion proteins, was loaded onto a glutathione-Sepharose 4B column (GE, Piscataway, NJ, USA) pre-equilibrated with buffer A (100 mM Tris-HCl, pH 8.0, 10 mM MgCl_2_, 1 mM DTT, and 1 mM PMSF). Non-specifically bound proteins were removed by washing with buffer A, and the bound fusion proteins with GST tags were eluted with buffer containing 50 mM Tris-HCl, pH 8.0, and 10 mM reduced glutathione.

### 3.5. Cleavage of the GST Tag

Recombinant GST-PtNAD-ME proteins bound to the column were digested by the addition of PreScission Protease (GE, Piscataway, NJ, USA) in elution buffer (50 mM Tris-HCl, pH 7.5, 150 mM NaCl, 1 mM EDTA, 1 mM DTT, and 0.01% Triton X-100) and incubated for 16 h at 4 °C. The desired recombinant proteins (each PtNAD-ME isoform) were eluted from the column and either used immediately for activity tests or stored at −80 °C for later use.

### 3.6. SDS-PAGE

Protein samples were analyzed by SDS-polyacrylamide gel electrophoresis (SDS-PAGE) as described by Laemmli [21] and stained with Coomassie brilliant blue R-250. The BCA protein assay (Pierce, Rockford, IL, USA) was used to determine the protein concentration.

### 3.7. Assay of NAD-ME Activity

NAD-ME activity was determined spectrophotometrically by monitoring NADH production at 340 nm. The standard reaction mixture contained 150 mM Mes/NaOH, 10 mM MnCl_2_, 4 mM NAD, and 10 mM malate in a final volume of 1 mL. One unit of enzyme activity is defined as the amount of enzyme resulting in the production of 1 μmol of NADH per minute. The reaction was started by the addition of NAD-ME. The optimum pH for NAD-ME was determined by measuring the oxidative decarboxylation of malate, which was described by Tronconi *et al.* [6]. When testing different compounds as possible inhibitors or activators of enzymatic activity, NAD-ME activity was measured in the presence of nonsaturating concentrations of malate while maintaining NAD concentrations at saturating levels, as described by Tronconi *et al.* [14]. The parameters were calculated by triplicate determinations, and the values are the average of three replicates differing by <5%.

### 3.8. Kinetic Studies on the Recombinant PtNAD-ME Proteins

The kinetic parameters were determined as described by Tronconi *et al.* [14]. Initial velocity studies were performed by varying the concentration of one substrate around its *K*_m_ while keeping other substrate concentrations at saturating levels. All kinetic parameters were calculated by triplicate determinations and adjusted to nonlinear regression. The concentrations of malate and NAD each varied between 0.008 and 16 mM. The values are the average of three replicates differing by <5%.

## 4. Conclusions

In this study, we have found that two α- and two β-NAD-ME genes exist in the genome of *Populus trichocarpa* and are expressed in various tissues. The characterization of recombinant *Populus* NAD-MEs *in vitro* indicates different enzymatic properties between α- and β-NAD-MEs. Also, the effects of metabolites on the activities of recombinant α-NAD-MEs are distinct from those of β-NAD-MEs. It suggests that *Populus* NAD-ME might be composed of dissimilar subunits where the α-subunit would contribute to catalysis and the β-subunit may have a regulatory role. Further, analysis of the deletion of α- or β-NAD-ME in future will reveal the function of PtNAD-ME in *Populus* biosynthetic pathway and respiratory metabolism.

## Supplementary Information



## Figures and Tables

**Table 1:** Summary of purification of four PtNAD-ME recombinant proteins from *E. coli* BL21 cells in 1 L cultures.

Recombinant enzyme	Purification step	Total protein (mg)	Total activity (U)	Specific activity a (U/mg)	Purification fold	Recovery (%)
PtNAD-ME1	Soluble fraction	537	107.4	0.2	1	100
	Cleavage of GST tag	2.3	78.2	34.0	170	72.9
PtNAD-ME2	Soluble fraction	569	170.7	0.3	1	100
	Cleavage of GST tag	2.7	109.4	40.5	142	64.0
PtNAD-ME3	Soluble fraction	529	2.1	0.004	1	100
	Cleavage of GST tag	1.3	1.6	1.2	300	74.3
PtNAD-ME4	Soluble fraction	533	1.6	0.003	1	100
	Cleavage of GST tag	1.4	1.3	0.9	300	78.7

a One unit of NAD-ME specific activity is defined as the amount of enzyme resulting in the production of 1 μmol of NADH per minute in the standard reaction mixture in a final volume of 1 mL.

**Table 2:** Kinetic parameters of four recombinant PtNAD-ME proteins purified from *E. coli*. The values are presented as the mean of triplicate determinations.

Parameters	*V*_max_ (μmol min^−1^·mg^−1^)		*K*_m_ (mM)		*k*_cat_ (s^−1^)		*k*_cat_/*K*_m_ (mM^−1^·s^−1^)	

	Malate	NAD	Malate	NAD	Malate	NAD	Malate	NAD
PtNAD-ME1	31.5	30.3	2.0	0.71	35.2	33.8	17.6	47.6
PtNAD-ME2	39.1	37.8	1.8	0.62	43.0	41.6	23.8	67.0
PtNAD-ME3	1.4	1.3	1.3	0.45	1.4	1.3	1.1	2.9
PtNAD-ME4	1.0	0.9	1.4	0.41	1.0	0.9	0.7	2.2
