# Supplementary material for: Enzymatic Properties of Populus α- and β-NAD-ME Recombinant Proteins
Source: Int J Mol Sci. 2013 Jun 24;14(7):12994–3004. doi: 10.3390/ijms140712994 (PMC3742170; doi:10.3390/ijms140712994)
Supplement: Supplementary file 1 [file ijms-14-12994-s001.pdf]

Supplementary Information

Table S1. The members of NAD-ME family in *Populus trichocarpa*.

JGI gene name	Assigned name	Location	Amino acid number	Protein ID
estExt_fgenesh4_pg.C_LG_II1218	*PtNADP-ME1*	LG_II:10105588-10114861	627	816506
eugene3.00400157	*PtNADP-ME2*	scaffold_40:1161255-1170200	627	591812
eugene3.00021450	*PtNADP-ME3*	LG_II:11890404-11897010	607	552135
estExt_Genewise1_v1.C_LG_XIV1110	*PtNADP-ME4*	LG_XIV:2174223-2181005	607	731028

Table S2. Homological analysis of amino acid sequences of PtNAD-ME proteins in *Populus trichocarpa*.

	PtNAD-ME1	PtNAD-ME2	PtNAD-ME3	PtNAD-ME4
PtNAD-ME1	100	96	66	65
PtNAD-ME2		100	66	65
PtNAD-ME3			100	92
PtNAD-ME4				100

Table S3. The sequences of primers for amplifying the CDS of *PtNAD-MEs*.

Gene	Primers for CDS (5'-3')
PtNAD-ME1	CACCATGCCGAATTTTTCCAATCAGA;<br>TCAATCTTTCTTATAGACCAGTGTTG
PtNAD-ME2	CACCATGTCAAATTTTTCCAATCAGAT;<br>TCAATCCCTCTTATAGACCAATGTTG
PtNAD-ME3	CACCATGTGGAGATTAGCGCGATGTGC;<br>CTATTTCTCATGAACAAGAGGGCTGT
PtNAD-ME4	CACCATGTGGAGGGTAGCGCGATTTGC;<br>TTATTTCTCATGAACAAGAGAGCTGTA

Table S4. The sequences of primers for real-time PCR of *PtNAD-MEs*.

Gene	Primers for semi-RT-PCR (5'-3')
PtNAD-ME1	AGCTCCGAAAACTCAGCCAG;<br>CTCAGATTTCAGGCGGTCAG
PtNAD-ME2	AGGATACCGTGAAATGGATGCT;<br>TTGACAACATGTTCGATCCCCA
PtNAD-ME3	CAGAAGGACATGGTGAGGCA;<br>ACTCGAGCCCTATTTCTCATGA
PtNAD-ME4	TGTGGCATATGTCATGCAGA;<br>AATATCGAGGCTGGATGCAG
PtActin2	GTTTCCAGGAATAGCTGATCG;<br>CCTCCGATCCAAACACTGTA

Table S5. Primers for amplifying the CDS for each mature PtNAD-ME proteins without signal peptides.

Protein	Peptide number	Primer sequences (5'-3')
PtNAD-ME1	592	GGATCCACTACATTGGAGGGTCACCG (*BamH* I site underlined)
		CTCGAGTCAATCTTTCTTATAGACCAGT (*Xho* I site underlined)
PtNAD-ME2	592	GGATCCACTACATTGGAGGGCCACCG (*BamH* I site underlined)
		CTCGAGTCAATCCCTCTTATAGACCAA (*Xho* I site underlined)
PtNAD-ME3	586	GGATCCACTGATATTCTCCATGATCC (*BamH* I site underlined)
		CTCGAGCTATTTCTCATGAACAAGAG (*Xho* I site underlined)
PtNAD-ME4	586	GGATCCGATATCCTTCATGATCCTTG (*BamH* I site underlined)
		CTCGAGTTATTTCTCATGAACAAGAGA (*Xho* I site underlined)

Figure S1. Exon/intron structures of the PtNAD-ME genes. Grayer boxes represent exons and black lines represent introns.

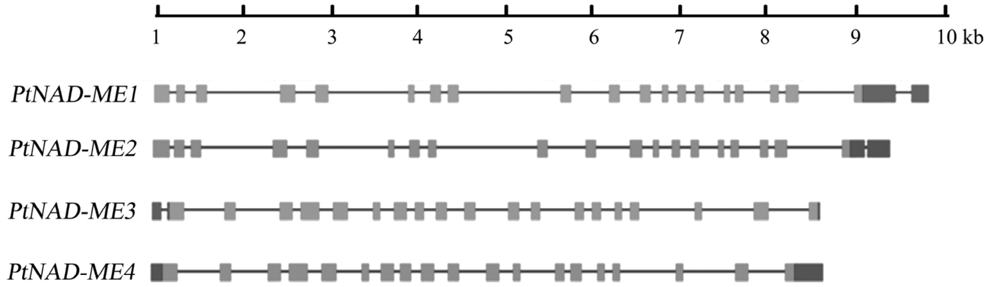

Figure S2. Multiple sequence alignment of full-length amino acid sequences of Populus NAD-ME proteins. Sequences were aligned using ClustalW2 program (http://www.ebi.ac.uk/Tools/msa/clustalw2). Consensus sequence is marked with *.

```
PtNAD-ME3      ---MWRLARCASSNLSRSLRR---------RFFSTAAIPAPCIIHKRGTDILHDPWFN 46
PtNAD-ME4      ---MWRVARFAASNVRSSSQR---------RFFSAAAIPGACIVHKRGADILHDPWFN 46
PtNAD-ME1      MPNFSNQIRASSSLIKRLQQRMTNPAALMQATRSFTTLEGHRPTIVHKRSLDILHDPWFN 60
PtNAD-ME2      MSNFSNQIRASSSLIKRLKQRMTNPAALMQATRHFTTLEGHRPTIVHKRSLDILHDPWFN 60
                  : .  * ::* :    :*           * *::     . *:***. *********

PtNAD-ME3      KDTGFPLTERDRLGLRGLLPPRVISFEQQYDRFMESYRSLEKNTQGQPYSVVSLAKWRIL 106
PtNAD-ME4      KDTGFPLTERDRLGLRGLLPPRVISFEQQYDRFMESYRSLEKNTQGQPYSVVSLAKWRIL 106
PtNAD-ME1      KGTAFSMTERDRLDIRGLLPPNVMSSEQQIQRFMVDLKRLEVQARDGPSDPNALAKWRIL 120
PtNAD-ME2      KGTAFSMTERDRLDLRGLLPPNVMTSEQQIQRFAADLKRLEVQARDGPSDPYALAKWRIL 120
               *.*.*.:******.:******.*:: *** :**  . : ** :::. * .  :*******

PtNAD-ME3      NRLHDRNETLYYRVLIDNIKDFAPIIYTPTVGLVCQNYSGLFRRPRGMYFSAKDKGEMMS 166
PtNAD-ME4      NRLHDRNETLYYRVLIDNIKDFAPIIYTPTVGLVCQNYSGLFRRPRGMYFSAKDKGEMMS 166
PtNAD-ME1      NRLHDRNETMYFKVLIANIEEYAPIVYTPTVGLACQNYSGLFRRPRGMYFSAEDRGEMMS 180
PtNAD-ME2      NRLHDRNETMYYQVLIANIEEYAPIVYTPTVGLVCQNYSGLFRRPRGMYFSAEDRGEMMS 180
               *********:*::*** **:::***:*******.*****************:*:*****

PtNAD-ME3      MIYNWPAQQVDMIVLTDGSRILGLGDLGVQGIGIPIGKLDMYVAAAGINPQKILPVMLDV 226
PtNAD-ME4      MIYNWPGQQVDMIVLTDGSRILGLGDLGVQGIGIPIGKLDMYVAAAGINPQRILPIMLDV 226
PtNAD-ME1      MVYNWPAEQVDMIVVTDGSRILGLGDLGVQGIGIAIGKLDLYVAAAGINPQRVLPVMIDV 240
PtNAD-ME2      MVYNWPAEQVDMIVVTDGSRILGLGDLGVQGIGIAIGKLDLYVAAAGINPQRVLPVMIDV 240
               *:****.:******:******************.*****:**********::**:*:**

PtNAD-ME3      GTNNQKLLEDPLYLGLRQPRLEGEEYLSIVDEFMEAVHTRWPKAIVQFEDFQMKWAFETL 286
PtNAD-ME4      GTNNQKLLEDPLYLGLRQPRLEGEEYLSIVDEFMEAVHTRWPKAIVQFEDFQMKWAFETL 286
PtNAD-ME1      GTNNEKLLKDPLYLGLQEHRLDGDEYIAVIDEFMEAVFTRWPHVIVQFEDFQSKWAFKLL 300
PtNAD-ME2      GTNNEKLLKDPLYLGLQENRLDGDEYIAVIDEFMEAVFTRWPHVIVQFEDFQSKWAFKLL 300
               ****:***:*******::  **:*:**::::*******.****:.******** ****: *

PtNAD-ME3      QRYRKRFCMFNDDIQGTAGVALAGLLGTVRAQGLPLSDFVNQKIVVVGAGSAGLGVLNMA 346
PtNAD-ME4      QRYRKRFCMFNDDVQGTAGVALAGLLGTVRAQGRPLSDFVNQKIVVVGAGSAGLGVLTMA 346
PtNAD-ME1      QRYRNAYRMFNDDVQGTAGVAIAGLLGAVRAQGRPMIDFPKQKIVVAGAGSAGIGVLNAA 360
PtNAD-ME2      QRYRNTYRMFNDDVQGTAGVAIAGLLGAVRAQGRPMIDFPKQKIVVAGAGSAGIGVLNAA 360
               ****: : *****:*******:*****:***** *: ** :*****.******:***. *

PtNAD-ME3      IQALSRMSGNNE---MAAKNKCYLLDKDGLITKERKNIDPAAAPFAKDLKDV--EGLREG 401
PtNAD-ME4      IQALSRMSGNNE---MAAKNQCYLLDKDGLITKERKNLDPAAAPFAKDIKDV--EGLREG 401
PtNAD-ME1      RKTMARMLGNNESAFESAGRQFWVVDAKGLITEERENIDLEALPFARKVEEASRQGLREG 420
PtNAD-ME2      RKTMARMLGNNESAFESAGRQFWVVDAKGLITEERENIDPEALPFARKVKEASRQGLREG 420
                :::** ****    :* .: :::* .****:**:*:*  * ***:.:::.   :*****

PtNAD-ME3      ASPFEVVKKLKPHVLLGLSGVGGVFNEEVLKAMRESDSTKPAIFAMSNPTMNAECTAADA 461
PtNAD-ME4      ASLLEVVKKLKPHVLLGLSGVGGIFNEQVLKAMRESDSPKPAIFSMSNPTMNAECNAADA 461
PtNAD-ME1      ASLAEVVREVKPDVLLGLSAVGGLFSKEVLEALKGSTSTRPAIFAMSNPTKNAECTPEEA 480
PtNAD-ME2      ASLAEVVREVKPDVLLGLSAVGGLFSNEVLEALKGSTSTRPAIFAMSNPTKNAECTPEEA 480
               **  ***:::**.******.***:*.::**:*:: * *.:****:***** ****..  :*

PtNAD-ME3      FKYAGPNIIFGSGSPFEDVDLGNGKVGHVNQANNMYLFPGIGLGTLLSGAHIITDGMLQA 521
PtNAD-ME4      FKHAGPNIVFASGSPFENVDLGNGKVGHVNQANNMYLFPGIGLGTLLSGAHVITDGMLQA 521
PtNAD-ME1      FSIVGDNIIFASGSPFKDVDLGNGHIGHCNQGNNMYLFPGIGLGTLLSGSRIISDGMLQA 540
               *.  .* **:*.*****::******::** **.*****************:::*:******

PtNAD-ME3      AAECLASYMTDEEIQNGILYPSIDSIRHITAEVGAAVLRAAVEEDLAEGHGEAGPRELKH 581
PtNAD-ME4      AAECLASYMTDEEIQKGILYPSIDSIRHITAEVGAAVVQAAVEEDLAEGHGDVGPRELKH 581
PtNAD-ME1      AAECLAAYMTEEEVLKGIIYPSTSRIRDITKEVAAAVVKEAIEEDLAEGYREMDARELRK 600
PtNAD-ME2      AAECLAEYMAEEEVLNGIIYPSTSRIRDITKEVAAAVVKEAIKEDLAEGYREMDARELQK 600
               ****** **::**: :**:*** .  **.** **.***:: *::******: : ..***::

PtNAD-ME3      MSKAETVAYVSRNMWFPVYSPLVHEK- 607
PtNAD-ME4      MSKEETVAYVMQNMWFPVYSSLVHEK- 607
PtNAD-ME1      LSQEEIEEYVKNNMWSPDYPTLVYKKD 627
```

Figure S3. SDS-PAGE analysis of the four recombinant Populus NAD-MEs without mitochondrial signal peptides expressed in *E. coli* BL21 cells. M, marker (91, 67, 43, 31, and 20 kDa); 1-8, 0 and 2h IPTG induction for cells with pGEX-6P-3-PtNAD-ME1, pGEX-6P-3-PtNAD-ME2, pGEX-6P-3-PtNAD-ME3, and pGEX-6P-3-PtNAD-ME4, respectively. Pound key indicate each GST-PtNAD-ME proteins.

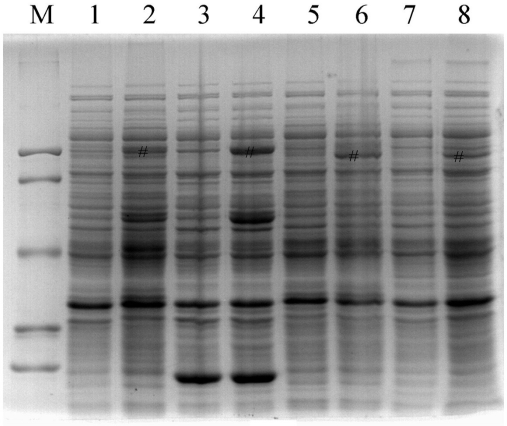

© 2013 by the authors; licensee MDPI, Basel, Switzerland. This article is an open access article distributed under the terms and conditions of the Creative Commons Attribution license (http://creativecommons.org/licenses/by/3.0/).
